# Beneficial effects of nicotine, cotinine and its metabolites as potential agents for Parkinson’s disease

**DOI:** 10.3389/fnagi.2014.00340

**Published:** 2015-01-09

**Authors:** George E. Barreto, Alexander Iarkov, Valentina Echeverria Moran

**Affiliations:** ^1^Department of Nutrition and Biochemistry, Pontificia Universidad JaverianaBogotá, D. C., Colombia; ^2^Center of Research in Biomedical Sciences, Universidad Autónoma de ChileSantiago, Chile; ^3^Research & Development Service, Bay Pines VA Healthcare SystemBay Pines, FL, USA; ^4^Research Service, James A Haley Veterans’ HospitalTampa, FL, USA; ^5^Department of Molecular Medicine, Morsani College of Medicine, University of SouthTampa, FL, USA

**Keywords:** Akt (PKB), synucleinopathies, beta-amyloid, cotinine, nicotine, Parkinson disease

## Abstract

Parkinson’s disease (PD) is a progressive neurodegenerative disorder, which is characterized by neuroinflammation, dopaminergic neuronal cell death and motor dysfunction, and for which there are no proven effective treatments. The negative correlation between tobacco consumption and PD suggests that tobacco-derived compounds can be beneficial against PD. Nicotine, the more studied alkaloid derived from tobacco, is considered to be responsible for the beneficial behavioral and neurological effects of tobacco use in PD. However, several metabolites of nicotine, such as cotinine, also increase in the brain after nicotine administration. The effect of nicotine and some of its derivatives on dopaminergic neurons viability, neuroinflammation, and motor and memory functions, have been investigated using cellular and rodent models of PD. Current evidence shows that nicotine, and some of its derivatives diminish oxidative stress and neuroinflammation in the brain and improve synaptic plasticity and neuronal survival of dopaminergic neurons. *In vivo* these effects resulted in improvements in mood, motor skills and memory in subjects suffering from PD pathology. In this review, we discuss the potential benefits of nicotine and its derivatives for treating PD.

## Introduction

Parkinson’s disease (PD) is the second most common neurodegenerative illness after Alzheimer’s disease (AD), and reaches a prevalence of 3% after 65 years of age (Jellinger, 2003). Parkinson’s disease is predominantly sporadic, and rarely familial. The familial form of the disease can develop due to single genetic mutations (Dexter and Jenner, 2013). Parkinson’s disease is characterized by the presence of Lewy bodies, mainly composed of alpha-synuclein fibrils, a depletion of dopamine (DA)-generating neurons in substantia nigra pars compacta (SNc) and ventral tegmental area (VTA) regions of the brain (Wirths and Bayer, 2003; Dexter and Jenner, 2013), that results in a decrease of DA levels in the striatum and frontal cortex regions of the brain (Thompson et al., 2005). Parkinson’s disease affects cognitive and motor abilities (Riedel et al., 2014), and progressively impairs sleep (Dos Santos et al., 2014), attention, and short-term memory, as well as visuospatial and executive functions (Liu et al., 2012; Conte et al., 2013; Rottschy et al., 2013; Zokaei et al., 2014). The dopaminergic deficit observed in PD patients seems to underlie the motor impairment symptoms such as hypokinesia, tremor and rigidity (Murer and Moratalla, 2011).

**Figure 1:**
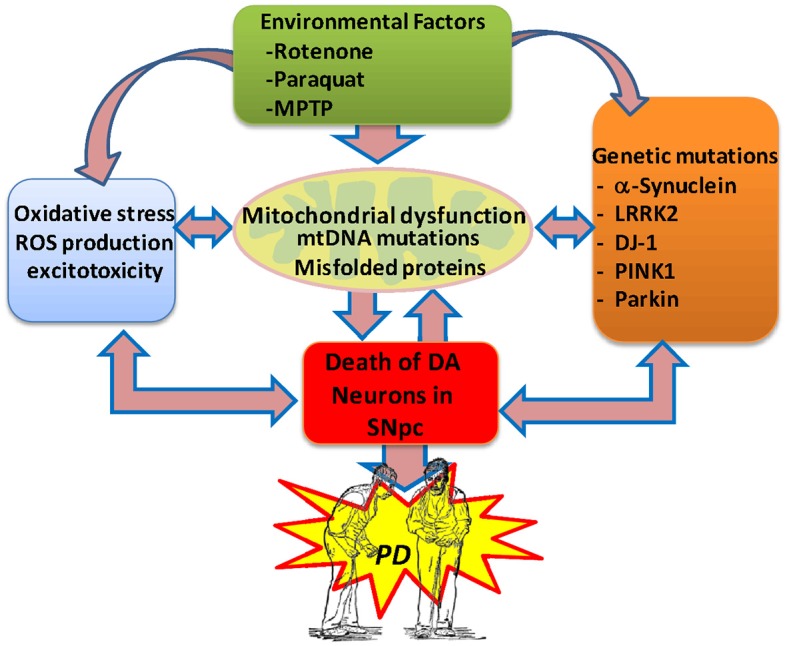
**Potential etiological factors of Parkinson’s disease**. Current evidence suggests that environmental factors and genetic risk factors provoking oxidative stress, excitotoxicity and mitochondrial dysfunction in the brain can lead to the degeneration of the midbrain dopaminergic system, resulting in PD. In PD, the generation of protein aggregates may disrupt the mitochondrial membrane potential and induce abnormal Ca^2+^ influx, impaired respiratory enzyme activities, reduced ATP generation and increased levels of reactive oxygen species. Also, abnormal release of cytochrome C from damaged mitochondria can trigger the activation of the apoptotic signaling cascades and the release of caspases, resulting in neuronal cell death. The generation of free radicals may then result in further damage to cellular macromolecules and organelles through nitrosylation, oxidation, and peroxidation, directly contributing to neuronal injury.

The cause of death of dopaminergic neurons is still a mystery; however, actual evidence is consistent with the idea that oxidative stress, mitochondrial dysfunction and neuroinflammation are the main factors involved in the etiology of PD (Büeler, 2010; Zuo and Motherwell, 2013; Camilleri and Vassallo, 2014; Celardo et al., 2014). It has been proposed that various genetic and environmental factors causing mitochondrial dysfunction result in abnormal accumulation of miscoded proteins and the generation of oxidative stress in the brain of subjects with PD (Bové and Perier, 2012; Cabezas et al., 2012, 2014; Perier and Vila, 2012; Dexter and Jenner, 2013; Trinh and Farrer, 2013; Zuo and Motherwell, 2013; Figure 1).

Mild cognitive impairment later progressing to dementia is commonly found in patients with PD (Aarsland et al., 2010; Hu et al., 2014). Dementia develops in approximately 80% of PD patients after 20 years with the condition (Cummings, 1988a,b; Hughes et al., 2000). New evidence also suggests that the level of alpha-synuclein in the cerebral spine fluid can predict the progression of cognitive decline but not of motor dysfunction in PD (Stewart et al., 2014). Dementia in PD is also associated with the accumulation in the central nervous system of protein aggregates such as β-amyloid peptide (Aβ) also present in other neurological conditions such as AD (Mandal et al., 2006; Kalaitzakis et al., 2008). The analysis of 30 PD cases from the United kingdom PD’s society showed that Aβ deposition in the striatum strongly correlated with dementia in humans suggesting that this accumulation is a contributing factor for the development of cognitive impairment and neurodegeneration in this condition (Mandal et al., 2006; Kalaitzakis et al., 2008). Subjects with PD and dementia show degeneration of several subcortical nuclei, including the cholinergic nucleus basalis of Meynert, the medial SN, and the noradrenergic locus coeruleus (LC; Shin et al., 2012). It has been postulated that the presence of secondary neuropathologies may further increase oxidative stress, decrease brain energy and enhance the degenerative process in the brain of subjects with PD (Sasaki et al., 1992; Kurosinski et al., 2002; Slawek et al., 2008).

## Oxidative stress, neuroinflammation, protein aggregation and brain energy deficits in Parkinson’s disease

Oxidative stress plays a key role, inducing damage to neuronal proteins, lipid and organelles in the SN of subjects with PD (Cabezas et al., 2012). Also as mentioned above abnormal aggregation and/or mutation of proteins such as beta amyloid, DJ-1, PTEN-induced putative kinase 1, parkin, leucine-rich repeat kinase 2 and α-synuclein have been observed in PD (el-Agnaf and Irvine, 2002; Nunomura et al., 2007; Alcalay et al., 2009). Families with autosomal dominant early-onset of familial PD (FPD) commonly present mutations in the α-synuclein gene A53T, A30P and E46K that accelerate α-synuclein aggregation (Corti et al., 2011; Roth, 2014). In addition to protein mutations, environmental factors such as pesticides, ethanol consumption, metals and the accumulation of somatic mitochondrial DNA mutations have been observed in the SNc during aging and PD (Zuo and Motherwell, 2013) that may negatively affect brain homeostasis leading to neurodegeneration (Perez et al., 2010; Perfeito et al., 2013; Roth, 2014). Protein aggregation may disrupt the mitochondrial membrane potential, impair respiratory enzyme activities and result in reduced ATP generation, energy deficits and increased levels of reactive oxygen species (O^2−^ and H_2_O_2_). In fact, energetic and antioxidant compounds are considered to have beneficial effects against PD (Klein and Ferrante, 2007; Sutachan et al., 2012). In turn, oxidative stress can also affect protein degradation by deteriorating the ubiquitin-proteasome system (Díaz et al., 2010; Perfeito et al., 2013). Furthermore, the release of cytochrome C from damaged mitochondria can trigger the activation of apoptotic cascades and neuronal cell death (Tatton et al., 2003). Also, free radical generation can promote nitrosylation, oxidation, and peroxidation of neuronal proteins, making the surviving neurons increasingly susceptible to further toxic insults (Barreto et al., 2011, 2014a).

Neuroinflammation and microglia activation seem to play an important role in PD pathogenesis (Tansey and Goldberg, 2010). Microglia represents about 10–15% of glial cells in the brain, and changes in the brain homeostasis induced by abnormal proteins aggregation, brain injury, and hypoxia, stimulate the release of reactive oxygen and nitrogen species, cytokines, and chemokines from these immune cells (Wyss-Coray and Mucke, 2002; McGeer and McGeer, 2004; Barreto et al., 2007, 2009, 2014b; Albarracin et al., 2012; Sutachan et al., 2012; Torrente et al., 2014). Microglia activation may have some beneficial effects helping with the clearance of aggregated proteins and cell debris. However, it is considered that persistent microglia activation further damages the brain and exacerbates the neurodegenerative process in PD.

A recent clinical study investigated the relationship between microglial activation, amyloid deposition, and glucose metabolism in 19 PD patients with and without dementia and 24 healthy controls subjects (Edison et al., 2013). Patients were evaluated using T1 and T2 magnetic resonance imaging (MRI) technology, and positron emission tomography (PET) scans, for the *in vivo* visualization of microglia activation glucose metabolism, and beta amyloid deposits by using the radiotracer [^11^C](R)-PK11195, [^18^F] fluoro-deoxy-glucose, and [^11^C]Pittsburgh compound B(PIB), respectively (Edison et al., 2013). Parkinson’s disease patients with dementia showed higher cortical microglia activation than healthy controls, a sign of neuroinflammation. In addition, a modest but consistent increase in Aβ deposition expressed as a slightly higher [^11^C] PIB uptake was observed in the cortex of PD patients. Microglia activation and energy deficits (glucose metabolism deficiency) are early events during the development of PD. Furthermore, the reduction in both glucose metabolism and microglia activation correlated with a decrease in mini-mental state examination score, a broadly used test of cognitive abilities. Altogether, this evidence suggests that these factors are relevant to the development of cognitive impairment and are good therapeutic targets for PD.

## Nicotine and its derivatives as therapeutic agents against Parkinson’s disease

In spite of over almost 200 years passed from its discovery, there are no drugs available to slow down or stop the progression of PD (Connolly and Lang, 2014). Motor symptoms in PD can be improved by N-methyl-NMDA blockers as well as using dopaminergic and anticholinergic compounds. However, the cognitive deficits are not substantially improved by the current therapeutic interventions (Lorenz et al., 2014). Furthermore, the most used anti-PD drug, levodopa, produces severe toxic effects such as restlessness, mental impairment, mood changes, and after prolonged use (3–5 years), dyskinesia (Simuni and Sethi, 2008). For this reason, the development of potential new therapeutic approaches is imperative.

In the PD brains, one of the regions more affected by the disease, the SNc, contains surviving neurons that may differ from the healthy ones by presenting Lewy bodies, neuromelanin and/or showing negative immunoreactivity for tyrosine hydroxylase (Faucheux et al., 2003; Hirsch et al., 2003a,b). These neurons may be a good target for neuroprotective or restorative therapeutic strategies, focused in decreasing oxidative stress and neuroinflammation. Studies revealed that cholinergic and dopaminergic systems work together to fine tune the striatum control of motor and cognitive functions. Then cholinergic dysfunction also may contribute to the neurotransmitter imbalance underlying PD (Zhou et al., 2003; Aosaki et al., 2010).

The striatum receives abundant cholinergic innervations. The neurons of the striatum express various types of muscarinic (mAChR) and nicotinic acetylcholine receptors (nAChRs), as well as DA receptors (Zhou et al., 2003). The cholinergic receptors modulate the dopaminergic system and are involved in motor and cognitive functions. Different subtypes of nAChRs are differentially expressed throughout the central nervous system and show diverse subunit composition including α3, α4, α5, α6 and α7, β2, α3 and β4 (Graham et al., 2002). In humans, the nicotinic receptors subtypes undergo changes during aging (Nordberg, 1994). Significant losses of nAChRs subunits α7 and α4 have been detected in the cortex from PD patients (Whitehouse et al., 1988; Burghaus et al., 2003).

Most immune cells such as B cells, monocytes and T cells express all subtypes of mAChRs (M1-M5), and the α3, α5, α7, α9, and α10 nAChR subunits and modulators of the AChRs can influence immunological response and inflammation (Gahring and Rogers, 2005; Carnevale et al., 2007). Nicotinic acetylcholine receptors regulate synaptic transmission and synaptic plasticity, in several regions of the brain including the midbrain DA centers. These receptors are ligand-gated Ca^2+^, Na^+^ and K^+^ channels, whose activation causes membrane depolarization and the increase of both intraneuronal calcium levels and neurotransmitter release probability. At the postsynaptic sites the activation of the nAChRs also stimulates cell signaling pathways promoting the expression of synaptic proteins mediating, at cellular level, higher cognitive functions such as attention, learning and memory and other cognitive functions (McKay et al., 2007). Furthermore, it has been shown that nAChRs activation prevents neurodegeneration by mechanisms involving the activation of pro-survival signaling factors such as phosphatidylinositol 3-kinase (PI3K), Akt and Bcl proteins in the brain (Kawamata and Shimohama, 2011).

In addition, modulators of the nAChRs such as nicotine, can have beneficial effects by stimulating cholinergic anti-inflammatory pathways (Gahring and Rogers, 2005). One of these pathways, involves the control of the immune response by the efferent vagus nerve, *via* the peripheral release of ACh that in turn activates the cholinergic receptors in the brain. Acetylcholine by stimulating the α7nAChR inhibits the activity of the pro-inflammatory neurothrophic factor kappa B (NFκB) in human macrophages and consequently the expression and release of cytokines such as the tumor necrosis factor (TNF) by these immune cells (Balakumar and Kaur, 2009). In fact, a knockout mouse showing a genetic deletion of the α7nAChR presented a significant increase in pro-inflammatory cytokines such as TNF-α, interferon-γ and IL-6 in spleen cells. This evidence showed that α7nAChRs are involved in the regulation of cytokine production (Fujii et al., 2007).

In rats, all dopaminergic neurons in the SNc express the α7nAChR (Yanagida et al., 2008). Since the α7nAChR activation stimulates signaling pathways that are anti-inflammatory and promote neuronal survival, the positive modulation of these receptors can be relevant for preventing neuroinflammation and promoting neuronal survival in the brain regions affected by PD. In this respect, several nicotinic receptors subunits such as the α7, α6β2 and α4β2 have been proposed as targets against PD (Quik and Wonnacott, 2011c).

Several epidemiological studies have shown that tobacco users have a lower incidence or severity of PD (Baron, 1996). A meta-analysis of four cohorts and 44 single-subject studies, showed a 40% decrease of risk of developing PD for smokers when compared to never smokers (Hernán et al., 2002). In addition, another study reported evidence suggesting that smoking prevented motor complications in a group of PD patients (De Reuck et al., 2005). Together with smoking, other natural products such as caffeine have shown to be preventative against PD. In one of these studies (Hernán et al., 2002), the epidemiological evidence on the association between cigarette smoking, coffee drinking, and the odds of developing PD was reviewed. Results for smoking were calculated based on 44 case-control and four cohort studies, and for coffee on eight case-control and five cohort studies. Compared with never smokers, the relative risk of PD was 0.59 for ever smokers, 0.80 for past smokers, and 0.39 for current smokers. Compared with non-exposed to caffeine controls, relative risk of PD was 0.69 for coffee drinkers. The relative risk per three additional cups of coffee per day was 0.75 in case-control studies and 0.68 in cohort studies. This meta-analysis showed strong epidemiological evidence supporting that in addition to nicotine also caffeine decreased the risk of PD (Chen et al., 2010).These findings have been confirmed by several large prospective studies for either coffee or cigarette consumption. Interestingly, other caffeine-containing beverages, such as black tea and Japanese and Chinese teas also reduced PD risk. Because numerous studies reported had lower sample size or limited longitudinal data, the relationship between tobacco consumption and lower risk to develop PD has been interpreted differently. For example, based in small case studies, some authors have suggested that PD patients display specific personality traits, such as lack of novelty seeking, that make them less prone to consume stimulants such as tobacco and coffee. A case-control study assessed the relationship between the ability to quit smoking and nicotine substitute use, in 1808 patients with PD and 1876 sex and age matched controls (Ritz et al., 2014). Data from this study revealed that PD patients quit smoking more easily than controls. The authors suggested that this finding was the result of a decreased sensitivity to nicotine in PD patients. Furthermore, they proposed that superior ability to quit smoking is an early sign of PD, similar to constipation, olfactory dysfunction, and sleep disorders. They also suggested that the positive effect of smoking revealed by epidemiologic studies was the result of reverse causation and not a protective effect of nicotine *per se*.

Albeit an interesting observation, newly published evidence, in agreement with previous studies, strongly supports the view that smoking was neuroprotective against PD. The analysis of longitudinal data from 305,468 participants of the NIH-AARP Diet and Health cohort, of whom 1662 had a PD diagnosis, clearly showed that the number of cigarettes and years of smoking inversely and significantly correlated with diminished odds of developing PD. Compared with non-smokers, the odds ratios were 0.78 for past smokers and 0.56 for current smokers. Furthermore, the time of smoking was linked to a lower risk of developing PD. When compared with non-smokers, the odds ratio among past smokers decreased with the smoking duration. Thus, the odds ratio for those who smoked more than 20 cigarettes per day per 1–9, 10–19, 20–29 and 30 years were 0.96, 0.78, 0.64 and 0.59, respectively. These studies strongly suggest that caffeine and nicotine are neuroprotective.

About the mechanism of neuroprotection, in addition to the mechanisms discussed, a new hypothesis proposes that both cigarette and coffee exert their effects by a completely different mechanism. The hypothesis states that tobacco and caffeine both exert their effects by changing the composition of the microbiota in the gut and consequently reducing intestinal inflammation since pro-inflammatory cytokines are also produced by enteric glial cells (EGC) in the gut (Derkinderen et al., 2014). According to this hypothesis, inflammation would promote α-synuclein aggregation within enteric neurons (EN). The aggregated protein thus may spread to the central nervous system via the vagal preganglionic innervation of the gut and the dorsal motor nucleus of the vagus (DMNV). After several years, the LC and the SN will become affected. Thus, the decrease of inflammation induced by nicotine and caffeine will decrease α-synuclein aggregation in enteric nerves, and its propagation to the brain. This effect will reduce neurodegeneration and reduce the risk of PD.

The neuroprotective effect derived from a general reduction of systemic inflammation is an interesting idea that merit further investigation. Certainly, we consider reasonable to propose that the anti-inflammatory effects of nicotine and nicotine-derived compounds is a key contributor to their neuroprotective effects in the brain.

The neuroprotective effect of tobacco has been mainly attributed to nicotine, a naturally occurring alkaloid from tobacco that consistently showed beneficial pro-cognitive effects in cellular and animal models of PD (Parain et al., 2003). The positive effect of nicotine on motor coordination and behavior has been attributed to its ability to increase DA availability and reduce the production of reactive oxygen species. Although, the detailed mechanism of neuroprotection is not well understood, the activation of nAChRs is considered the main mechanism of action of nicotine against PD (Budzianowski, 2013).

The derivative of synthetic heroin, 1-Methyl-4-phenyl-1,2,3,6-tetrahydropyridine (MPTP), elicits PD-like symptoms in experimental primates and rodents and drug addicts, giving a clear clue that environmental factors may be involved in the development of this disease (Quik et al., 2006a,b). In fact, acute exposure to MPTP induces striatal neurodegeneration in the brain of primates, mimics many of the characteristics of PD pathology. Chronic nicotine treatment protected against MPTP-induced synaptic plasticity and dopaminergic dysfunction, as well as prevented striatal neurodegeneration in non-human primates (Quik et al., 2006a,b). This beneficial effect of nicotine was also observed in another model of PD, the methamphetamine-induced neurotoxicity in rodents (Maggio et al., 1997). Interestingly, they found that (-)-nicotine’s actions correlated with an increase in the expression of the neurotrophic factors, the basic fibroblast growth factor-2 and the brain-derived neurotrophic factor in the rat striatum. The effect of (-)-nicotine on the induction of fibroblast growth factor 2 was prevented by the nAChR antagonist mecamylamine (Maggio et al., 1997).

This evidence further supported the view that the neuroprotective effects of nicotine were the result of nAChRs stimulation, which promotes DA release and increases the survival of dopaminergic neurons by activating anti-apoptotic factors. These results are coherent with the idea that nicotine may prevent PD by stimulating the expression of neuroprotective signaling factors in the brain.

Also, the effects of nicotine and cotinine on L-DOPA-induced dyskinesias have been investigated in L-DOPA-treated unilateral 6-hydroxydopamine-lesioned rats, a well-established model of dyskinesia. By using this model, it was found that daily treatment with nicotine (0.1–0.2 mg/kg, for 4 or 10 days) but no cotinine, reduced the abnormal involuntary movements. The effect was not observed after acute nicotine administration. Several days of mecamylamine (1.0 mg/kg) injection also ameliorated dyskinesias to a comparable extent to nicotine, suggesting that the latter may have exerted its effects by desensitizing nAChRs. Another study tested the protective potential of ABT-107, a highly selective agonist of the α7 nAChRs in rats lesioned by infusion of 6-hydroxydopamine (6-OHDA) into the medial forebrain (Bordia et al., 2014) were implanted with mini pumps containing ABT-107 or nicotine. They found that 2-week treatment with ABT-107 or nicotine in rats, improved two measures of Parkinsonism, the contralateral forelimb use and adjusted stepping. These treatments also significantly improved striatum integrity and basal DA release from lesioned striatum, as well as nicotine-stimulated DA release mediated via α4β2 and α6β2 nAChRs. In summary, epidemiological studies consistently have shown a lower risk of PD among smokers suggesting that this association is causal (Mellick et al., 2006; O’Reilly et al., 2009).

One latest study investigated the effect of two β2 nAChR agonists, ABT-089 and ABT-894, on levodopa-induced dyskinesias (LIDs) in 1-MPTP-lesioned monkeys. Monkeys were administered levodopa/carbidopa until they were stably dyskinetic. Each set had a vehicle-treated group, a nAChR agonist-treated group, and a nicotine-treated group as a positive control. For the experiments with nAChR agonist, two groups of monkeys were used, one group has been previously treated with nAChR drugs (nAChR drug-primed), and a second group was nAChR drug-naive. Both groups were treated with the partial agonist ABT-089 orally for 5 days a week 30 min before levodopa, with each dose given for 1–5 weeks. ABT-089 decreased LIDs by 30% to 50% compared with vehicle-treated monkeys. Nicotine reduced LIDs by 70% in a parallel group. After 4 weeks of washout, the effect of the full agonist ABT-894, was assessed on LIDs in both groups. ABT-894 reduced LIDs by 70%, similar to nicotine. Both drugs acted equally well at α4β2 and α6β2 nAChRs. Tolerance did not develop for the time periods tested (range, 3–4 months). The nAChR drugs did not induce significant side effects, and were effective to manage LIDs in PD.

This evidence suggests that positive modulators of the nAChRs, such as cotinine, can be excellent drugs to minimize LID in PD (Zhang et al., 2014).

Together, this evidence supports the view that α7 nAChR agonists may improve motor behaviors associated with PD by enhancing striatal dopaminergic function.

On the other hand, evidence suggests that nicotine binds and inhibits the fibrillation of both α-synuclein (Hong et al., 2009) and Aβ (Zeng et al., 2001), thus preventing its toxic effects. The presynaptic protein, α-synuclein is broadly expressed in the brain and its aggregation is a pathological hallmark of several neurodegenerative diseases, including PD (el-Agnaf and Irvine, 2002; Schmid et al., 2013). The effects of several tobacco compounds including anabaseine, cotinine, hydroquinone, nicotine and nornicotine on α-synuclein fibrillation have been studied using atomic force microscopy, gel electrophoresis, size exclusion chromatography–high performance liquid chromatography and thioflavin assays. Using these techniques, they discovered that nicotine and, at a lower extent, hydroquinone, inhibited α-synuclein fibrillation by stabilizing the soluble oligomeric forms of the protein. These data indicated that nicotine in addition to its cholinergic effects may prevent the generation of toxic protein aggregates of α-synuclein in the brain. Similar experiments showed an inhibition of Aβ peptide aggregation by cotinine *in vitro* and *in vivo* (Echeverria et al., 2011). On the other hand, research evidence from a separated study, suggests that a decrease in protein misfolding induced by these compounds can also be due to a reduction of protein aggregation by their direct binding to α-synuclein. This study investigated the binding of caffeine, curcumin, and nicotine to α-synuclein by nano pore analysis. They found that these compounds bind to α-synuclein causing large conformational changes in it. This changes may affect the aggregation of this protein resulting in another neuroprotective effect. Interestingly caffeine and nicotine seems to bind to α-synuclein simultaneously (Tavassoly et al., 2014).

## Nicotine metabolites

Nicotine is metabolized by the enzymes cytochrome P450 2A6 (CYP2A6), UDP-glucuronosyl transfease and flavin-containing monooxygenase. The metabolism of nicotine is greatly affected by genetic factors influencing CYP2A activity, diet components such as grape fruit, age, sex, the use of estrogen, pregnancy and kidney disease, and other medications. People treated with nicotine have sustained brain levels of cotinine (Bergen and Caporaso, 1999), thus cotinine may underlie at least in part the beneficial effects of nicotine in PD. Nicotine is metabolized in the liver to six primary metabolites, cotinine, nicotine *N*′-oxide, nornicotine, hydroxynicotine, anbaseine (Benowitz et al., 1994). In addition, Nicotine 2′-hydroxylation produces 4-(methylamino)-1-(3-pyridyl)-1-butanone with 2′-hydroxynicotine as an intermediate. 2′-hydroxynicotine also yields nicotine-Δ^1′(2′)^-iminium ion. 4-(methylamino)-1-(3-pyridyl)-1-butanone that is further metabolized to 4-oxo-4-(3-pyridyl) butanoic acid and 4-hydroxy-4-(3-pyridyl) butanoic acid 9 (Hecht et al., 2000). Nicotine *N*′-oxide, (4–7% of nicotine metabolism) is synthesized by a flavin-containing monooxygenase 3 (Cashman et al., 1992; Benowitz and Jacob, 1994). Following the systemic administration of nicotine, its metabolites cotinine, nornicotine, and norcotinine can be found in the brain (Neurath, 1994). Currently, the effect(s) of these metabolites and newly synthetized derivatives against neurodegeneration is under intense scrutiny.

## Cotinine

Cotinine is a component of the tobacco leaves and the main metabolite of nicotine, with about 70–80% of nicotine being converted to cotinine in a two-step process that is dependent on the genetic background (Benowitz et al., 1996, 2011). The first step, catalyzed by CYP2A6, involves the synthesis of nicotine-Δ^1′(5′)^-iminium ion, which is in equilibrium with 5′-hydroxynicotine. The second step, catalyzed by an aldehyde oxidase, produces cotinine (Benowitz et al., 2009). In addition, six main metabolites of cotinine have been reported in humans: 3′-hydroxycotinine (McKennis et al., 1963; Neurath, 1994), 5′-hydroxycotinine (also called allohydroxycotinine) that exists in tautomeric equilibrium with the open chain derivative 4-oxo-4-(3-pyridyl)-*N*-methylbutanamide (Neurath, 1994), cotinine *N*-oxide, cotinine methonium ion, cotinine glucuronide, and norcotinine (also called dimethylcotinine) (Benowitz and Jacob, 1993, 1994, 1997).

The total clearance of nicotine averages about 1200 ml min^−1^. Clearance of nicotine is decreased in the elders (age >65) compared to adults (Molander et al., 2001). Neonates have diminished nicotine metabolism, and have a nicotine half-life three to four times longer than in adults (Dempsey et al., 2000). The metabolism of cotinine is much slower than that of nicotine. Clearance of cotinine and (3′*R*, 5′*S*)-trans-3′-hydroxycotinine is slow and averages about 45 ml min^−1^ and 82 ml min^−1^, respectively. Distinctive from nicotine, cotinine does not have addictive or major negative side effects in humans and its half-life has shown to be similar in neonates, older children, and adults (Dempsey et al., 2000, 2013). The clearance of nicotine is more influenced by changes in hepatic blood flow than cotinine whose clearance is more dependent on enzymatic metabolism. Neonates have only slightly lower amounts of CYP2A6, CYP2D6, and CYP2E1 protein in liver, whereas the CYP2B6 amount is clearly lower in neonates than both adults and older children.

Several studies in animal models of the disease have shown that cotinine is a memory enhancer that prevents cognitive impairment induced by different neurological and mental conditions including chronic psychological stress, antagonism of the NMDA receptors, agonism of the DA receptors and the development of AD-like pathology in rodents (Moran, 2012). Cotinine also showed neuroprotective effects against Aβ toxicity in primary neurons (Burgess et al., 2012) and glutamate in PC12 cells (Buccafusco and Terry, 2003). More importantly, chronic treatment of transgenic AD mice with cotinine, before full pathology development, prevented memory loss and reduced plaque aggregation in the brain of mice (Echeverria et al., 2011; Echeverria and Zeitlin, 2012). Furthermore, twice the daily dose of cotinine (5 mg/kg) administered after full development of the pathology for a few months also improved working and reference memory, reduced plaque pathology and increased the expression of the postsynaptic density protein 95 (PSD95) in the hippocampus of the AD mice (Grizzell et al., 2014). The positive effect of cotinine on memory seems not to be restricted to rodents, as pro-cognitive effects of cotinine have been also shown in monkey models of schizophrenia and aging (Buccafusco and Terry, 2003, 2009; Terry et al., 2012). New evidence showed that cotinine preserves synaptic density by preventing synaptic loss induced by chronic stress (Moran, 2012; Grizzell et al., 2014).

Since the cholinergic and dopaminergic systems influence each other in the brain (Aosaki et al., 2010; Havekes et al., 2011), it is expected that cholinergic agonist/modulators may affect the dopaminergic system. An ideal treatment for PD should reduce damage of dopaminergic neurons in midbrain; in this respect interestingly, it has been shown that both nornicotine, and cotinine evoke DA release from striatal and cortical slices in rats, respectively (Dwoskin et al., 1999). *In vitro* studies showed that cotinine evoked (^3^H)DA- release at concentrations similar to the ones estimated to be reached in the brain of smokers ( >1 μM, EC_50_ = 30 μM ). Cotinine was less potent than either nicotine or nornicotine in inducing the release of DA (Dwoskin et al., 1999). These effects were mediated by the nAChRs as were blocked by their antagonists, mecamylamine and DHBE. On the other hand, nornicotine, similarly to nicotine, activated the neural mechanism responsible for behavioral sensitization. Since behavioral sensitization would be dependent on the activation of DA systems, and nornicotine evokes DA release, these results suggest a significant role for nornicotine in the behavioral sensitization produced by nicotine.

Regarding potential therapeutic targets of nicotine-derived compounds, the nAChRs subtypes, α6 β2 β3 and α6 α4 β2 β3, play a key role in modulating dopaminergic function in the mesolimbic and nigrostriatal dopaminergic pathways. Since these systems are critically involved in behaviors linked to the control of movement, these receptors may represent ideal therapeutic targets against nigrostriatal damage and for the treatment of PD (Quik and Wonnacott, 2011c; Quik et al., 2011b, 2012a). It is reasonable to speculate that cotinine and/or nicotine, by targeting these receptors, may be beneficial against PD (Quik et al., 2012b). In recent years, we and others have proposed that cotinine functions as a positive allosteric modulator (PAM) of α7 nAChRs (Echeverria and Zeitlin, 2012). Positive allosteric modulator compounds enhance the response and inhibit the desensitization of the nicotinic receptor by its agonists. Also, by using [^3^H] DA release assays and ligand-binding autoradiography in monkey’s striatum, it was discovered that cotinine functionally interacts with both α4β2 and α3α6β2 subtypes of nicotinic receptors in the caudate promoting DA release. This effect could prove useful for the future development of therapies for PD (O’Leary et al., 2008).

**Figure 2:**
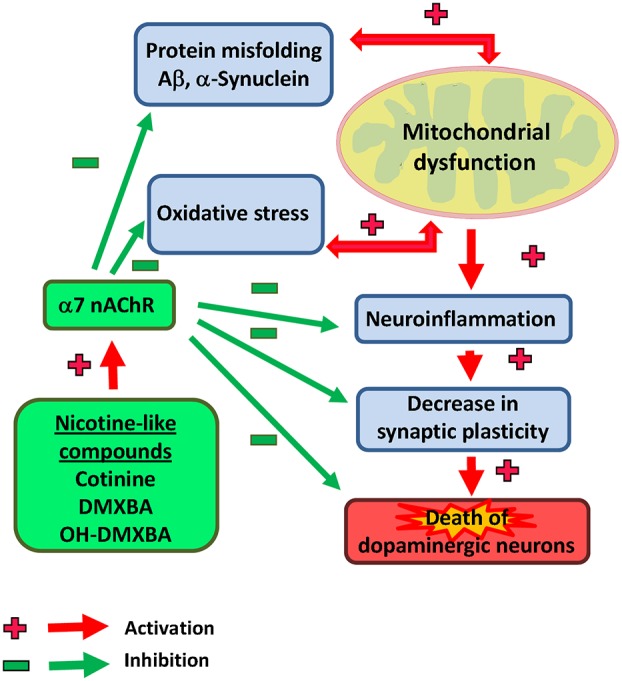
**Illustrative view of the potential mechanisms of action of nicotinic analogs to prevent Parkinson’s disease**.

In addition, based on previous reports showing an anti-inflammatory effect of the nAChRs modulation (Kalra et al., 2002, 2004), we hypothesize that cotinine may also be beneficial reducing the neuroinflammation process observed in PD brains. For example, an *in vitro* study showed an anti-inflammatory effect of nicotine by inhibiting microglia cell activation (Guan et al., 2014). Interestingly, cotinine suppresses the release of oxygen free radicals from neutrophils (Srivastava et al., 1989) and augments PI3K-dependent anti-inflammatory pathways in human monocytes (Rehani et al., 2008; Figure 2).

## Anabaseine and nicotine

Anabasine is a tobacco alkaloid, occuring as a mixture of enantiomers in wild tree tobacco (*Nicotinana glauca*). Anabasine is similar in structure to anabaseine, an alkaloid occuring in certain animal venoms are toxins, but lacks the imine double bond of anabaseine. These two compounds are modulators of the α7nAChRs. The relative agonistic potencies of the agonists on human fetal nicotinic neuromuscular receptors order: anabaseine >> S-anabasine > R-anabasine. Anabaseine stimulates α7nAChRs currents at higher rates than nicotine (65% response) and equivalent to acetylcholine in the receptor expressed in *Xenopus* oocytes (Kem et al., 1997). Several nicotinic agonists derived from anabaseine have been characterized. One of these compounds, DMXBA [3-(2,4-dimethoxybenzylidene)-anabaseine; code name GTS-21] is an antagonist of the α4/β2 receptors and a selective agonist of the α7 receptors. DMXBA has shown promising characteristics in phase I studies (Kem, 2000). One of these studies indicated that DMXBA improved cognitive abilities and was safe when administered orally to healthy young male subjects. DMXBA is a partial agonist of the α7 receptors, is much less toxic than nicotine, and does not affect autonomic and skeletal muscle systems at doses, which enhance cognitive behavior. In addition, DMXBA is neuroprotective against β-amyloid toxicity or NGF deprivation. In comparison to full agonists, partial agonists only elicit a partial response still after full binding to the α7 receptors. Because of this limited activity, partial agonists are less toxic and addictive than full agonist (Shi et al., 2013).

A recent study investigated the effects of DMXBA, a selective α7nAChR agonist (Briggs et al., 1997), in a rat 6-OHDA-induced PD-like model (Suzuki et al., 2013). This model is based in the fact that 6-OHDA when microinjected into the nigrostriatal pathway of rats selectively induces the apoptosis of dopaminergic neurons. Injection of 6-OHDA increased microgliosis and astrogliosis markers in the SNc of rats. Dopaminergic neurodegeneration and gliosis was markedly inhibited by co-administration of DMXBA. This agonist prevented neuronal loss by a mechanism dependent on the α7nAChR, since it was abolished by its selective receptor’s antagonist methyllycaconitine since microglia in the SNc also expresses α7nAChR in both resting and activated states (Briggs et al., 1995). It is reasonable to propose that anabaseine by acting on the nAChRs in neurons and glia, may prevent PD development by reducing apoptosis and neuroinflammation (Suzuki et al., 2013).

Similar neuroprotective effects of nicotine in the model of rotenone-induced PD-like pathology have been previously reported in rats (Costa et al., 2001). Both rotenone and 6-OHDA are strong inhibitors of the mitochondrial complex I, a protein complex located at the mitochondrial inner membrane and protruding into the matrix (Meredith et al., 2008; Kawamata and Shimohama, 2011). This neuroprotective effect was inhibited by mecamylamine, a general nAChR antagonist; the α7 specific competitive antagonist, α-bungarotoxin, and dihydro-β-erythroidine dehydropyridine (DHPE) a potent antagonist of the α4β2 neuronal nAChRs (Luetje et al., 1990). These findings suggest that nicotine-induced neuroprotective effects are mediated via nAChRs, especially through α7 and α4β2 receptors. They also showed that nicotine prevented rotenone-induced dopaminergic neuronal death via the PI3K. Despite these encouraging effects, nicotine is not an optimal therapeutic drug because it represents a health risk for its toxicity and addictive effects. Moreover, evidence that long-term nicotine exposure both depressed DA release in the nucleus accumbens of non-human primates and decreased the expression of α6β2 nAChRs in the same brain region of rats, further weakens the enthusiasm of using nicotine as a therapy against PD (Perez et al., 2012, 2013). About the relationship between PD and nAChRs, a recent report investigating 596 patients suffering from PD and 369 control subjects showed that lower expression of the β3 nAChR subunit was associated with nicotine dependance in PD. They found a significant increase of the c.-57G allele of the CHRNB3 gene codifying for β3 nAChR among PD patients when compared to controls. The authors suggest that the CHRNB3c.-57 A > G change affects CHRNB3 gene promoter activity and that this allele is associated with PD and smoking. They also concluded that nicotine may be especially beneficial in PD patients with specific genotypes (Bar-Shira et al., 2014).

## Safety of nicotine use

Several forms of administration of nicotine has been tested in humans including transdermal patches, gums, lozenges, tablets, inhalers, electronic cigarettes, and nasal sprays. Nicotine replacement therapies are considered safer than tobacco use, having much less harmful effects than cigarette smoke (Schneider et al., 2001; Caldwell et al., 2012; Gupta and Babu, 2013; Palazzolo, 2013; Sanford and Goebel, 2014). The results of several pilot clinical studies investigating the effect of nicotine on PD’s symptoms suggest that nicotine or drugs modulating the nAChRs have potential for treating PD (Kelton et al., 2000; Quik et al., 2011a). Unfortunately, due to the small size of the cohorts and disparities of the protocols used, these studies have not given conclusive results of nicotine’s efficacy in decreasing motor or non-motor symptoms, best daily dosage or method of administration (Thiriez et al., 2011).

Despite the experimental evidence suggesting that this alkaloid may have different effects depending on the concentration reached in the body, nicotine is still considered a toxic and addictive compound. High doses of nicotine may hasten tumors and atheromas growth by promoting angiogenesis, and neovascularization (Dasgupta and Chellappan, 2006; Pillai and Chellappan, 2012). Additionally, the activation of nAChR by nicotine may increase the occurrence of choroidal neovascularization as observed in aged smokers with macular degeneration (Davis et al., 2012). However, low doses of nicotine seem to have different effects. For example, nicotine used in the form of nasal spray did not affect blood pressure and hematologic parameters in smokers (Benowitz et al., 2002). Similarly, transdermal nicotine did not increase the incidence of myocardial ischemia (Benowitz and Gourlay, 1997). Neither, nicotine induced exacerbation of arrhythmia and/or myocardial ischemia in those with preexisting coronary artery disease (Benowitz and Gourlay, 1997). Substantial evidence supports the view that at low concentrations (1–30 nM), nicotine enhances the release of vasodilators and increases the survival of endothelial cells. Conversely, at higher concentrations nicotine is toxic and has deleterious effects on the vasculature (Yu et al., 2009).

As an alternative option, the reduction of the dose of nicotine needed for therapeutic use is currently being investigated. For example, a recent study assessed the effect of nicotine-encapsulated poly (lactic-co-glycolic) acid (PLGA) nanoparticles on MPTP-induced neurotoxicity and neuroinflammation in a mouse model of PD (Tiwari et al., 2013). As previously shown, MPTP reduced tyrosine hydroxylase immunoreactivity and DA levels, and increased several markers of neuroinflammation such as microglial activation, and the expression of the inducible nitric oxide synthase, metallothionein-III, heme oxygenase 1. 1-Methyl-4-phenyl-1,2,3,6-tetrahydropyridine also increased the levels of protein markers of apoptosis, such as p53 and caspase-3, as well as markers of oxidative stress including nitrite and lipid hydroperoxidase in the brain. However, nicotine was neuroprotective and improved the survival of tyrosin hydroxylase immunoreactive neurons in the brain of mice. Both nicotine and nicotine-PLGA nanoparticles normalized the levels of these biomarkers, but nanotization of nicotine improved its bioavailability and efficacy in reducing the indicators of oxidative stress and apoptosis (Tiwari et al., 2013). This evidence suggests that this new delivery approach may facilitate the use of nicotine or its metabolites against PD.

The negative effects of nicotine can be counteracted and several pharmacological agents have shown promises to prevent the deleterious effects of nicotine such as the cardiovascular abnormalities associated with the chronic nicotine exposure. Some evidence obtained in rodent models suggests that agents or natural extracts with antioxidant properties, such as melatonin and oil onion may prevent the nicotine-induced myocardial injury by activating antioxidant defense and reducing oxidative stress (Helen et al., 1999; Baykan et al., 2008; Rodella et al., 2012). Likewise, the administration of vitamin E reduces both oxidative stress and endothelial dysfunction induced by cigarette smoking (Heitzer et al., 1999).

In addition to these natural compounds, other drugs have been tested to counteract the potential oxidative stress induced by nicotine. For example, angiotensin, by activating type 1 receptors, is a major activator of the nicotinamide adenine dinucleotide phosphate-oxidase (NADPH-oxidase) complex, involved in oxidative stress and inflammatory processes. Several studies have demonstrated that hyperactivation of the renin-angiotensin system in the SN and striatum may exacerbate oxidative stress by stimulating the NADPH-oxidase activity, and the microglial inflammatory response (Garrido-Gil et al., 2013; Labandeira-García et al., 2014). These effects may certainly contribute to the degeneration of dopaminergic neurons in those regions. Thus, angiotensin receptor blockers and angiotensin converting enzyme (ACE) inhibitors have been proposed as protective drugs against oxidative stress and inflammation derived from smoking and also in PD pathology (Wright and Harding, 2012).

Although a comprehensive understanding of the mechanisms of neuroprotection by nicotine-like compounds is still in progress (see Table 1), it is generally accepted that the activation of nAChRs is a required step. Future clinical studies directed towards establishing the potential for nicotine metabolites to treat neurological and psychiatric disorders for which the positive modulation of the nAChRs have shown beneficial effects such as AD, depression, post-traumatic stress disorder, schizophrenia and Tourette’s syndrome.

**Table 1:** **Effect of Nicotine and some nicotine derivatives on Parkinson disease pathology**.

Drug	Dopamine levels	Striatum Integrity/neuroprotection	Cognitive abilities/Synaptic plasticity	Motor functions	References
Nicotine	(Long-term)	+			Costa et al. (2001)
		+			Guan et al. (2014)
		+			Maggio et al. (1997)
		+			Parain et al. (2003)
	−				Perez et al. (2012)
		+	+	+	Quik et al. (2006a,b)
		+		+	Tiwari et al. (2013)
		+		+	Yanagida et al. (2008)
Cotinine		+	+		Buccafusco and Terry (2003)
			+		Buccafusco and Terry (2009)
		+			Burgess et al. (2012)
	+				Dwoskin et al. (1999)
			+		Echeverria et al. (2011)
			+		Grizzell et al. (2014)
		+			Parain et al. (2003)
			+		Terry et al. (2012)
ABT-107		+		+	Bordia et al. (2014)
ABT-089/		+		+	Zhang et al. (2014)
ABT-894
DMXBA			+		Kem (2000)
		+		+	Suzuki et al. (2013)

## Concluding remarks

Preclinical studies showed that nicotine’s metabolites that are positive cholinergic modulators show pro-cognitive, anti-inflammatory and neuroprotective properties associated with the positive modulation of the cholinergic and dopaminergic systems. Derivatives of nicotine such as cotinine have great potential to become effective agents to prevent and alleviate neurological symptoms developed in subjects with Parkinsonism. It is surprising the absence of funding for the clinical development of these compounds, as they could be therapeutic solutions which have been lying in front of our eyes for hundreds of years, waiting for development. The savings in public health costs will definitely overcome the costs of the clinical testing of these compounds and will positively affect the society as a whole by increasing the quality of life of millions of our elder citizens.

## Author’s contributions

All authors participated in drafting and revising the manuscript for intellectual content.

## Conflict of interest statement

Drs. George Barreto and Alexander Iarkov have no actual or potential conflict of interests concerning the research in the present paper. Valentina Echeverria Moran is the inventor of a pending patent application from the University of South Florida and the Veterans Affairs administration (US 20100104504).

## Abbreviations

Aβ: beta-amyloid
AChE: acetylcholinesterase
AD: Alzheimer’s disease
Akt (PKB): protein kinase B
DA: Dopamine
DMXBA: 3-(2,4-dimethoxy) benzylidene-anabaseine dihydrochloride
FPD: familial PD
GSK3β: glycogen synthase kinase 3β
FS: forced swim
iNOS: the inducible nitric oxide synthase
MAOIs: monoamine oxidase inhibitors
MT: metallothionein
NFκB: neurothrophic factor kappa B
nAChRs: nicotinic acetylcholine receptors
NMDA: N-methyl-D-aspartate
PAM: positive allosteric modulator
PD: Parkinson’s disease
PI3K: phosphatidylinositol 3-kinase
PIB: Pittsburgh compound B
PSD95: postsynaptic density protein 95
SNc: substancia nigra pars compacta
TNF: tumor necrosis factor
VTA: ventral tegmental area.
